# Aerobic Capacity Reference Data in 3816 Healthy Men and Women 20–90 Years

**DOI:** 10.1371/journal.pone.0064319

**Published:** 2013-05-15

**Authors:** Henrik Loe, Øivind Rognmo, Bengt Saltin, Ulrik Wisløff

**Affiliations:** 1 K.G. Jebsen Center of Exercise in Medicine at Department of Circulation and Medical Imaging, NTNU, Trondheim, Norway; 2 Valnesfjord Rehabilitation Center, Valnesfjord, Norway; 3 Copenhagen Muscle Research Centre, University Hospital, Copenhagen, Denmark; Universidad Europea de Madrid, Spain

## Abstract

**Purpose:**

To provide a large reference material on aerobic fitness and exercise physiology data in a healthy population of Norwegian men and women aged 20–90 years.

**Methods:**

Maximal and sub maximal levels of VO_2_, heart rate, oxygen pulse, and rating of perceived exertion (Borg scale: 6–20) were measured in 1929 men and 1881 women during treadmill running.

**Results:**

The highest VO_2max_ and maximal heart rate among men and women were observed in the youngest age group (20–29 years) and was 54.4±8.4 mL·kg^−1^·min^−1^ and 43.0±7.7 mL·kg^−1^·min^−1^ (sex differences, p<0.001) and 196±10 beats·min^−1^ and 194±9 beats·min^−1^ (sex differences, p<0.05), respectively, with a subsequent reduction of approximately 3.5 mL·kg^−1^·min^−1^ and 6 beats·min^−1^ per decade. The highest oxygen pulses were observed in the 3 youngest age groups (20–29 years, 30–39 years, 40–49 years) among men and women; 22.3 mL·beat^−1^±3.6 and 14.7 mL·beat^−1^±2.7 (sex differences, p<0.001), respectively, with no significant difference between these age groups. After the age of 50 we observed an 8% reduction per decade among both sexes. Borg scores appear to give a good estimate of the relative exercise intensity, although observing a slightly different relationship than reported in previous reference material from small populations.

**Conclusion:**

This is the largest European reference material of objectively measured parameters of aerobic fitness and exercise-physiology in healthy men and women aged 20–90 years, forming the basis for an easily accessible, valid and understandable tool for improved training prescription in healthy men and women.

## Introduction

Evidence supports a strong inverse association between cardiorespiratory fitness and all-cause mortality [1]–[4]. Therefore, in order to increase the individual’s fitness level, different types of exercise training are used both in prevention and treatment of cardiovascular and life-style related disease [5]. In order to prescribe a proper prevention- or treatment program either, the more reliable, individually cardiopulmonary exercise testing is needed [6], [7], or one can rely upon previously established reference material. Most previous studies measuring cardiorespiratory fitness tend to use peak oxygen uptake (VO_2peak_), indirect methods, estimation by equation, selected populations and/or small sample sizes [8]–[15]. Previously established variables suitable for exercise prescription such as oxygen uptake, heart rate, Watts and Borg scale scores, are mostly based upon small sample sizes or poorly described populations [6], [16]–[23]. The aim of this study was to establish a large reference material of empirical cardiorespiratory fitness and exercise-physiology data in healthy men and women aged 20–90 years in order to provide an easily accessible, valid and understandable tool for improved exercise training prescription.

## Methods

### Participants

The HUNT 3 fitness study is the third wave of the Nord-Trøndelag Health Studies (www.ntnu.edu/hunt). Data were collected between October 2006 and June 2008. The entire population >20 years of age (n = 94194) were invited to participate, 54% (n = 50821) accepted. The HUNT 3 Fitness Study was designed to acquire reference material for submaximal and maximal cardiorespiratory parameters in a healthy population. Exclusion criteria were cancer, cardiovascular disease, obstructive lung disease and use of blood pressure medication. Participants also had to be cleared in a brief medical interview. Based upon self-reported information, 30513 candidates presented as suitable for VO_2max_ testing. Out of these, 12609 candidates resided in the 3 municipals selected for VO_2max_ testing, and 5633 of them volunteered to participate. These 3 locations were chosen due to geographical location to minimize travel distance for participants. 4621 candidates completed a VO_2max_ test, whereas 3816 tests were considered to have reached the true VO_2max_.

### Ethics Statement

The study was approved by the Regional committee for medical research ethics (2012/1228/REK midt), the Norwegian Data Inspectorate and the National Directorate of Health, and is in compliance with the Helsinki declaration. Written informed consent was obtained from all participants.

### VO_2max_ and Heart Rate

An individualized graded protocol [24] was used for measuring VO_2max_ (Cortex MetaMax II, Cortex, Leipzig, Germany). Prior of starting the testing procedure several MetaMax II apparatus were tested against Douglas-bag and iron lung (Cortex, Leipzig, Germany). Two MetaMax II apparatus were returned to Cortex due to unstable recordings (both ventilation and carbon dioxide analysis) and replaced by two new apparatus that were tested and found both reliable and valid. Hence, all MetaMax apparatus used in the project were both valid and reliable. Test-retest correlation of oxygen-uptake for the tested-personnel in the project was 0.99, p<0.001 and coefficient of variation was 1.8%. Bland-Altman plot were constructed where differences in two tests of maximal oxygen uptake (and sub maximal) from each person (test-1 minus test-2) were plotted against the average of the two tests ((test-1+test-2)/2). Average difference was −0.04 ml·kg^−1^·min^−1^ and standard deviation of the difference was 1.0. Therefore one can expect that 95% of all observations are at the average ((test-1+test-2)/2) ±2 standard deviations. Thus, we can expect values to vary between −0.04 −2⋅1 to −0.04+2⋅1 if we test maximal oxygen uptake twice in the same person within a short time period. Velocity and inclination of the test treadmills were calibrated prior to testing.

The MetaMax II was calibrated prior to the first test each day using a standard two-point gas calibration procedure recommended by the manufacturer. The calibration includes measurements of ambient air and a gas mix of known content (15.03% O_2_ and 4.98% CO_2_ in N_2_), a calibration of the Triple-V volume transducer with a calibrated 3 L syringe (Calibration syringe D, Sensormedics, CareFusion, San Diego, CA, USA), and barometric pressure control. Volume calibration was implemented every third test and the two-point gas calibration every fifth. Before each test the ambient room air was routinely checked. Heart rate was measured by radio telemetry (Polar S610i, Polar Electro Oy, Kempele, Finland). Body mass was measured using the weighing scale Model DS-102 (Arctic Heating AS, Nøtterøy, Norway). All participants had a treadmill familiarization period of 8–10 minutes during warm-up. They were instructed to avoid grabbing handrails if not necessary. The individualized warm-up workload determined the initial velocity/inclination on the subsequent treadmill test. Candidates wore a face mask (Hans Rudolph, Germany) linked to the MetaMax II. When participant maintained a stable oxygen uptake >30seconds, velocity (0.5–1.0 kmh-1) or inclination (1–2%) were increased. Increased workload was preferably obtained with increased velocity and keeping a fixed slope angle. If a participant was unable to increase velocity, inclination was increased. The average velocity and slope during test protocol were 6.8±2.2 km·h^−1^ (range 2–17 km·h^−1^) and 10.0±1.6% (range 2–16%), respectively. Tests were terminated when candidates were exhausted or reached a VO_2_ plateau that remained stable despite increased work load [25], i.e. VO_2_ did not increase more than 2 mL·kg^−1^·min^−1^ despite increased work load.

### Ventilatory Equivalent

We calculated the ventilatory equivalent (V_E_·VO_2_^−1^) at VO_2max_. The ventilatory equivalent describes the fraction of minute ventilation (V_E_) to oxygen uptake (VO_2_), hence, the higher the value the more ineffective is the V_E_. At higher levels of increasingly harder submaximal workloads, a disproportionate increase in V_E_ relative to VO_2_ occurs. This heralds an increasingly more inefficient V_E_.

### Questionnaire-based Information

Physical activity index score (PAI) was calculated from replies in a self-administered questionnaire that consisted of 3 questions. Question 1: “How frequently do you exercise?” with response alternatives “Never” (0); “Less than once a week” (0); “Once a week” (1); “2–3 times a week” (2.5); “Almost every day” (5). Question 2: “If you exercise as frequently as once or more times a week: How hard do you push yourself?” with response alternatives “I take it easy without breaking a sweat or losing my breath” (1); “I push myself so hard that I lose my breath and break into sweat” (2); “I push myself near exhaustion” (3). Question 3: “How long does each session last?” with the following response options “Less than 15 minutes” (0.1); “15–29 minutes” (0.38); “30 minutes to 1 hour” (0.75); “More than 1 hour” (1.0). The numbers in brackets, corresponding to each subject’s response to the 3 questions above, were multiplied to calculate the physical activity index score. An index score in the range 0.05–1.50 was considered to signify low activity, an index score in the range 1.51–3.75 was interpreted as medium activity and a score in the range 3.76–15.0 signified high activity. The index score is previously established as valid and reliable [26].

### Borg Scale of Perceived Exertion and VO_2_ at 2 Sub Maximal and Maximal Workload

Candidates were asked to state their subjective rating of perceived exertion (Borg scale) at the end of 3 different levels. Borg scale visualizes work load intensity, denoted by numbers 6–20 [22], with a proportional relation between increased rating of perceived exertion and the reported Borg scale number. Level 1: The individual initial workload for the test was determined during warm-up. All individuals reached a stable VO_2_ and heart rate after 3 minutes at the first submaximal work load. Level 2: Treadmill gradient was elevated by 2% or velocity increased 1 km·h^−1^. After 1–2 minutes at this sub maximal workload steady state was obtained. The maximum level is described previously.

### Watts

Workload in watts was calculated at the 3 described workloads. Calculations were based on treadmill slope gradient, velocity and body mass input in Cortex MetaMax II (Cortex, Leipzig, Germany). Minimum slope gradient was 2% and mean slope gradient at maximum workload was 10.4±1.4%.

### Statistical Analysis

Parametric analysis was used based on the large sample size. QQ-plots supported the assumption of normally distributed data. Descriptive data are presented as arithmetic mean ± standard deviation. An Independent-Samples T test was used for establishing level of significance between sexes and age groups. Linear regression, with 95% confidence interval, was used to illustrate associations between physiological parameters. All statistical tests were two-sided. SPSS 16.0 (Statistical package for Social Sciences, Chicago; Illinois, USA) and GraphPad Prism 4.01 (GraphPad Software, San Diego, California, USA) were used to analyze data. Correlations were done using data from Level 1, Level 2 and Level 3 (maximum) as described above. A p-value of <0.05 was considered statistically significant.

## Results

Overall VO_2max_ was 3.19±0.90 L·min^−1^ or 41.3±9.2 mL·kg^−1^·min^−1^ (range 18.6–76.5 mL·kg^−1^·min^−1^, Table 1). Women had 18.7% (p<0.001) lower VO_2max_ than men (37.0±7.5 mL·kg^−1^·min^−1^ vs. 45.4±8.9 mL·kg^−1^·min^−1^). Maximal oxygen pulse was 34% lower (p<0.001) in women than men (14.0±2.6 mL·beat^−1^ vs. 21.1±3.8 mL·beat^−1^). Maximal workload at VO_2max_ was 33% lower (p<0.001) in women compared with men (121±24 W vs. 181±36 W). There were no significant sex differences in maximal heart frequencies or Borg scores at termination of the test (Table 1).

**Table 1:** Physical and physiological characteristics of participants in the HUNT 3 Fitness study.

	All	Male	Female
	(n = 3678)	(n = 1929)	(n = 1881)
Age (years)	46.7±13.1	47.5±13.1	45.8±13.0
Body mass(kg)	77.3±13.7	85.3±11.1	69.2±11.0
Height (cm)	172.9±9.0	179.5±6.4	166.1±5.8
VO_2max_ (L·min^−1^)	3.19±0.90	3.83±0.72	2.53±0.49
VO_2max_ (mL·kg^−1^·min^−1^)	41.3±9.2	45.4±8.9	37.0±7.5
VO_2max_ (mL·kg^−0.75^·min^−1^)	122.0±27.8	137.3±25.6	106.2±20.2
O_2_ pulse (mL·beat^−1^)	17.7±4.9	21.1±3.8	14.0±2.6
R (CO_2_·VO_2_^−1^)	1.14±0.05	1.14±0.05	1.14±0.05
f_c_ (beats·min^−1^)	181±14	182±14	181±14
Work load (Watts)	151±43	181±36	121±24
Borg	18±1	18±1	18±1
Physical activity index	3.41±2.88	3.31±2.99	3.52±2.76

Data is presented as arithmetic mean ±SD. VO_2max_: maximal oxygen uptake, O_2_ pulse: oxygen uptake per heartbeat, CO_2_: Carbon dioxide, R: respiratory exchange ratio, f_c_: cardiac frequency, workload: treadmill exercise load, BORG: subjective perception of fatigue (6–20), Physical activity index: A weighted product score between training- intensity, duration and frequency.

The highest VO_2max_ and maximal heart rate for both sexes were observed in the age group 20–29 years. Among men and women in this age group VO_2max_ were 54.4±8.4 mL·kg^−1^·min^−1^ and 43.0±7.7 mL·kg^−1^·min^−1^ (sex differences, p<0.001) with corresponding heart rates of 196±10 beats·min^−1^ and 194±9 beats·min^−1^ (sex differences, p<0.05). In both sexes VO_2max_ and maximal heart rates were approximately 8% (≈3.5 mL·kg^−1^·min^−1^) and 3.5% (6 beats·min^−1^) lower per increased decade, respectively. The Physical Activity Index (PAI) scores were also highest for both sexes in this age group. For men and women PAI scores were 4.64±4.04 and 3.96±3.25, respectively (no significant sex differences), which are considered to indicate a high physical activity level. All other age groups, regardless of sex, had PAI scores in the range 2.67–3.70, which are considered to indicate medium activity [26] (Table 2).

**Table 2:** Physiological variables in the HUNT 3 Fitness study stratified by sex and age groups.

	Male	Female
	20–29 years	
	(n = 199)	(n = 215)
VO_2max_ (L·min^−1^)	4.32±0.71	2.78±0.46
VO_2max_ (mL·kg^−1^·min^−1^)	54.4±8.4	43.0±7.7
VO_2max_ (mL·kg^−0.75^·min^−1^)	162.1±23.7	121.7±20.1
EqVO_2max_ (V_E_·VO_2max_^−1^)	33.9±4.0	34.1±5.3
Body mass (kg)	80.1±10.6	65.5±10.4
Height (cm)	181±6	166±6
R (CO_2_·VO_2_^−1^)	1.15±0.05	1.15±0.05
f_c_ (beats·min^−1^)	196±10	194±9
Workload (Watts)	200±39	128±24
BORG	19±1	18±1
PAI	4.64±4.03	3.96±3.25
	**30–39 years**	
	(n = 324)	(n = 359)
VO_2max_ (L·min^−1^)	4.22±0.63	2.75±0.48
VO_2max_ (mL·kg^−1^·min^−1^)	49.1±7.5	40.0±6.8
VO_2max_ (mL·kg^−0.75^·min^−1^)	149.2±21.0	114.9±18.1
EqVO_2max_ (V_E_·VO_2max_^−1^)	33.2±3.7	34.1±4.6
Body mass (kg)	86.8±12.1	69.7±11.5
Height (cm)	180±6	168±5
R (CO_2_·VO_2_^−1^)	1.15±0.05	1.15±0.05
f_c_ (beats·min^−1^)	190±9.5	188±11
Workload (Watts)	197±33	128±23
BORG	18±1	18±1
PAI	3.15±3.23	3.36±2.73
	**40–49 years**	
	(n = 526)	(n = 493)
VO_2max_ (L·min^−1^)	4.03±0.61	2.65±0.44
VO_2max_ (mL·kg^−1^·min^−1^)	47.2±7.7	38.4±6.9
VO_2max_ (mL·kg^−0.75^·min^−1^)	143.3±21.4	110.3±18.1
EqVO_2max_ (V_E_·VO_2max_^−1^)	33.6±4.3	34.6±4.5
Body mass (kg)	86.4±11.5	69.9±11.2
Height (cm)	180±7	167±6
R (CO_2_·VO_2_^−1^)	1.15±0.05	1.15±0.05
f_c_ (beats·min^−1^)	184±11	182±11
Workload (Watts)	189±33	125±22
BORG	18±1	18±1
PAI	3.00±2.73	3.70±2.66
	**50–59 years**	
	(n = 466)	(n = 428)
VO_2max_ (L·min^−1^)	3.65±0.59	2.36±0.37
VO_2max_ (mL·kg^−1^·min^−1^)	42.6±7.4	34.4±5.7
VO_2max_ (mL·kg^−0.75^·min^−1^)	129.5±21.1	98.7±15.0
EqVO_2max_ (V_E_·VO_2max_^−1^)	33.9±4.9	33.9±4.2
Body mass (kg)	86.4±10.3	69.6±10.2
Height (cm)	179±6	165±6
R (CO_2_·VO_2_^−1^)	1.14±0.05	1.14±0.05
f_c_ (beats·min^−1^)	177±12	176±11
Workload (Watts)	173±28	117±23
BORG	18±1	18±1
PAI	3.24±2.70	3.52±2.80
	**60–69 years**	
	(n = 300)	(n = 240)
VO_2max_ (L·min^−1^)	3.30±0.55	2.16±0.33
VO_2max_ (mL·kg^−1^·min^−1^)	39.2±6.7	31.1±5.1
VO_2max_ (mL·kg^−0.75^·min^−1^)	118.5±19.4	89.6±13.4
EqVO_2max_ (V_E_·VO_2max_^−1^)	34.9±5.0	34.1±4.4
Body mass (kg)	84.7±9.9	70.4±10.9
Height (cm)	179±6	165±5
R (CO_2_·VO_2_^−1^)	1.14±0.05	1.12±0.05
f_c_ (beats·min^−1^)	171±13	169±12
Workload (Watts)	160±33	110±23
BORG	17±1	17±2
PAI	3.22±2.58	3.19±2.57
	**+70 years**	
	(n = 76)	(n = 53)
VO_2max_ (L·min^−1^)	2.81±0.50	1.85±0.35
VO_2max_ (mL·kg^−1^·min^−1^)	35.3±6.5	28.3±5.2
VO_2max_ (mL·kg^−0.75^·min^−1^)	105.3±18.5	80.3±13.8
EqVO_2max_ (V_E_·VO_2max_^−1^)	36.0±5.2	34.9±4.4
Body mass (kg)	80.2±9.6	66.2±11.2
Height (cm)	176±6	162±6
R (CO_2_·VO_2_^−1^)	1.12±0.05	1.11±0.03
f_c_ (beats·min^−1^)	163±15	165±16
Workload (Watts)	140±31	97±26
BORG	17±1	16±2
PAI	3.46±2.92	2.67±1.92

Data is presented as arithmetic mean ± SD. VO_2max_: maximal oxygen uptake, EqVO_2max_: ventilatory equivalents, R: respiratory exchange ratio, f_c_: cardiac frequency, Workload: treadmill exercise load, BORG: subjective perception of fatigue (6–20), PAI: physical activity index: A weighted product score between training- intensity, duration and frequency.

We observed an EqVO_2max_ of 33.9±4.0 and 34.1±5.3 among men and women, aged 20–29 years, respectively. Generally no differences were found between sexes and age groups, except from a 3% (p<0.05) higher EqVO_2max_ in females aged 30–39 and 40–49 years compared to corresponding groups of males. Additionally, EqVO_2max_ increased by 3% (p<0.05) between the two most senior male groups (Table 2).

The highest maximal oxygen pulse was observed in the 3 youngest age groups (20–49 years) for both sexes, with no significant difference between these age groups. Women in these age groups had 33% lower (p<0.001) oxygen pulse compared with men (14.7±2.7 mL·beat^−1^ vs. 22.3±3.6 mL·beat^−1^). In the subsequent age groups an approximately 8% reduction in oxygen pulse per decade was observed among both sexes (Table 3).

**Table 3:** O_2_-pulse in the HUNT 3 Fitness study stratified by intensity levels, sex and age groups.

	Male			Female		
	Level 1	Level 2	Maximal	Level 1	Level 2	Maximal
**20–29 years**						
	(n = 121)	(n = 106)	(n = 199)	(n = 131)	(n = 111)	(n = 215)
O_2_-pulse (mL·beat^−1^)	17.5±5.5	18.4±4.0	22.1±4.0	11.8±2.4	12.6±2.2	14.4±2.4
VO_2_ (mL·kg·min^−1^)	34.6±8.0	40.4±8.1	54.4±8.4	29.3±6.2	33.9±6.5	43.0±7.7
%VO_2max_	63.7±12.3	72.7±10.6		68.1±11.0	76.6±9.6	
%f_cmax_	80.7±8.8	87.8±5.4		85.0±8.3	90.7±6.4	
Workload (watts)	109±24	126±23	200±39	78±15	91±14	128±24
**30–39 years**						
	(n = 176)	(n = 166)	(n = 324)	(n = 247)	(n = 221)	(n = 359)
O_2_-pulse (mL·beat^−1^)	17.5±2.9	18.3±2.9	22.3±3.6	12.2±2.5	12.9±2.6	14.7±2.7
VO_2_ (mL·kg·min^−1^)	29.8±5.5	34.2±5.7	49.1±7.7	27.2±5.3	31.0±5.5	40.0±6.8
%VO_2max_	62.1±10.4	71.0±10.1		68.5±10.4	77.7±9.9	
%f_cmax_	78.9±7.4	86.0±6.4		83.3±6.9	90.3±5.8	
Workload (watts)	110±21	124±20	197±33	77±16	91±18	128±23
**40–49 years**						
	(n = 351)	(n = 334)	(n = 526)	(n = 347)	(n = 320)	(n = 493)
O_2_-pulse (mL·beat^−1^)	18.4±3.9	19.1±4.0	22.1±3.6	12.3±2.5	13.0±2.54	14.6±2.6
VO_2_ (mL·kg·min^−1^)	30.7±6.8	34.8±7.1	47.2±7.7	26.5±5.03	30.2±5.9	38.4±6.9
%VO_2max_	65.1±10.6	73.7±10.4		70.1±10.4	79.0±9.8	
%f_cmax_	78.6±7.7	85.7±7.3		83.1±7.1	90.0±6.0	
Workload (watts)	107±21	123±22	189±33	74±15	88±16	125±22
**50–59 years**						
	(n = 343)	(n = 314)	(n = 466)	(n = 354)	(n = 311)	(n = 428)
O_2_-pulse (mL·beat^−1^)	17.8±3.3	18.5±3.3	20.7±3.6	11.8±2.1	12.3±2.2	13.4±2.2
VO_2_ (mL·kg·min^−1^)	28.5±6.0	32.1±6.1	42.6±7.4	24.1±4.0	27.1±4.3	34.4±5.7
%VO_2max_	67.6±10.9	75.6±10.6		72.0±10.1	80.2±9.8	
%f_cmax_	78.3±7.7	85.2±7.3		82.2±7.1	88.4±6.4	
Workload (watts)	99±20	117±21	173±28	67±17	79±18	117±23
**60–69 years**						
	(n = 258)	(n = 239)	(n = 300)	(n = 236)	(n = 204)	(n = 240)
O_2_-pulse (mL·beat^−1^)	17.1±3.3	17.5±3.4	19.3±3.4	11.7±2.5	11.9±2.1	12.9±2.3
VO_2_ (mL·kg·min^−1^)	26.6±5.5	29.6±5.4	39.2±6.7	22.9±3.8	25.5±4.3	31.1±5.1
%VO_2max_	66.4±11.0	76.5±11.0		74.8±9.9	81.9±9.2	
%f_cmax_	77.9±8.5	84.6±8.0		83.8±8.4	89.3±7.4	
Workload (watts)	88±20	104±21	160±33	57±17	70±18	110±23
**+70 years**						
	(n = 95)	(n = 86)	(n = 76)	(n = 83)	(n = 71)	(n = 53)
O_2_-pulse (mL·beat^−1^)	15.3±3.1	15.8±3.3	17.2±3.2	10.1±2.2	10.6±2.1	11.4±2.4
VO_2_ (mL·kg·min^−1^)	24.3±5.2	26.9±6.1	35.3±6.5	20.7±4.1	22.8±4.6	28.3±5.2
%VO_2max_	70.7±10.9	77.8±11.0		74.5±10.4	81.8±9.0	
%f_cmax_	80.9±7.7	86.2±8.0		84.2±7.2	90.0±7.7	
Workload (watts)	67±25	83±27	140±31	43±18	57±19	97±26

Data is presented as arithmetic mean ± SD. O_2_-pulse: oxygen pulse, VO_2_: oxygen uptake, %f_cmax_: percent of maximum heart frequency, Workload: treadmill exercise load.

### Rating of Perceived Exertion, %VO_2max_ and %maximal Heart Rate

As can be seen from Table 4, rating of perceived exertion reported as Borg Score fairly well estimate the relative exercise intensity expressed as percent of maximal heart rate and percent of VO_2max_. Data also show that there may be sex differences in these relationships in the lowest intensities corresponding to Borg below 16. For example the actual exercise intensity for men and women that report to exercise at Borg 6–9 corresponds to 75.8% (CI: 74.5–77.1) and 79.1% (CI: 77.6–80.5) of maximal heart rate, respectively (sex differences, p<0.05). Furthermore, Borg 13–15 corresponds to a heart rate of 84.7% (CI: 84.3–85.1) for men and 88.4% (CI: 88.0–88.7) for women (sex differences, p<0.001). The same discrepancies apply to %VO_2max_ in corresponding Borg range. At Borg Score above 16 there were no sex or age group differences, with the exception of an age group difference between the 50–59 years and the 60–69 years group in both percent of maximal heart rate (p<0.01) and percent of VO_2max_ (p<0.05).

**Table 4:** Relationships between perceived exertion, VO_2max_ and f_cmax_ in the HUNT 3 fitness study.

	All			Male			Female		
Borgscale	% f_cmax_	95% CI	N	% f_cmax_	95% CI	N	% f_cmax_	95% CI	N
6–9	77.3	76.4–78.3	253	75.8	74.5–77.1	136	79.1	77.6–80.5	117
10–12	79.9	79.5–80.3	1475	78.1	77.6–78.6	813	82.2	81.6–82.7	662
13–15	86.6	86.3–86.7	3017	84.7	84.3–85.1	1459	88.4	88.0–88.7	1558
16–18	98.1	97.9–98.3	1775	98.1	97.8–98.4	862	98.2	97.9–98.5	913
19+	99.9	99.9–100	1216	99.9	99.7–100	560	100	99.98–100	656
**Borgscale**	**%VO_2max_**	**95% CI**	**N**	**%VO_2max_**	**95% CI**	**N**	**%VO_2max_**	**95% CI**	**N**
6–9	61.9	60.6–63.3	253	60.1	58.2–62.1	136	64.0	62.3–65.7	117
10–12	66.8	66.3–67.3	1475	64.7	64.1–65.4	813	69.3	68.6–70.0	662
13–15	76.7	76.3–77.1	3017	74.4	73.8–75.0	1459	78.8	78.3–79.4	1558
16–18	96.4	96.0–96.8	1775	96.5	96.0–97.1	862	96.3	95.8–96.8	913
19+	99.9	99.5–100	1216	99.8	99.5–100	560	99.9	99.9–100	656

Borgscale: subjective perception of perceived exertion (6–20), CI: confidence interval, %f_cmax_: percent of maximal heart frequency, %VO_2max_: percent of maximal oxygen uptake.

### VO_2_, Heart Rate, Watt and Physical Activity Index

**Figure 1:**
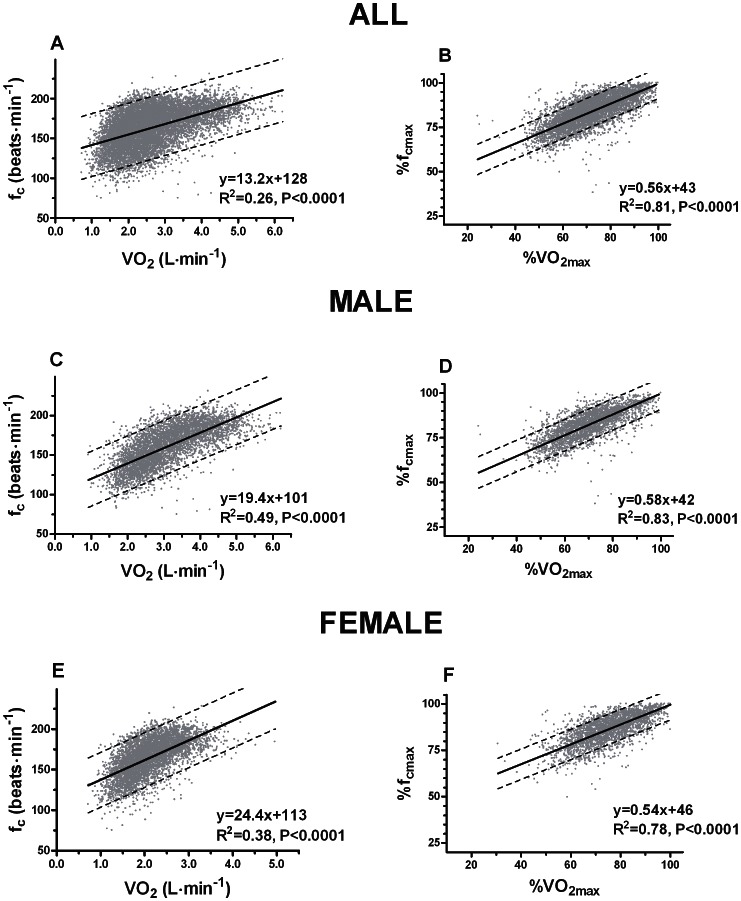
Correlations between oxygen uptake (VO_2)_ and heart rate (f_c_).

Figure 1 displays the relationship between VO_2_ and heart rate. The overall correlation for VO_2_ (L·min^−1^) and heart rate was found to be moderate (r = 0.51, p<0.0001). Stratified by sex, this association became stronger; males r = 0.70 (p<0.0001), female r = 0.61 (p<0.0001). The association between percent VO_2_ and percent maximal heart rate was strong; all r = 0.89 (p<0.0001), male r = 0.90 (p<0.0001), female r = 0.87 (p<0.0001).

**Figure 2:**
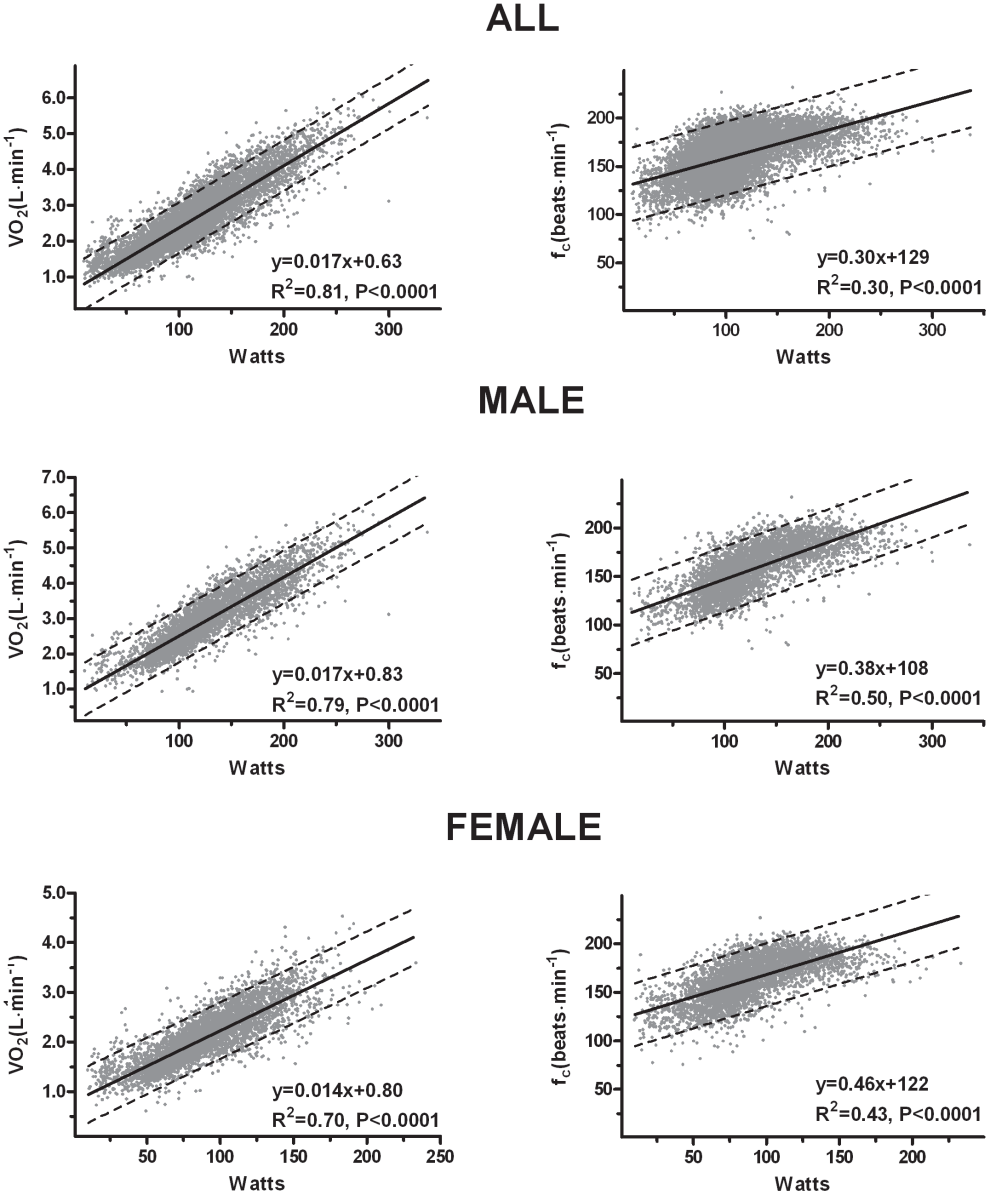
Correlations between workload (Watts) and oxygen uptake (VO_2_) and correlations between Watts and heart rate (f_c_).

Figure 2 demonstrates the strong association between VO_2_ (L·min^−1^) and treadmill workload (Watts), and the correlation between watts and heart rate; VO_2_ vs. Watts: all r = 0.90 (p<0.0001), male r = 0.89 (p<0.0001), female r = 0.84 (p<0.0001). Heart rate vs. Watts: Viewing the full sample size a moderate correlation was observed between treadmill workload (Watts) and heart rate (r = 0.55, p<0.0001). Good correlations were observed when the data was stratified by sex; male r = 0.71 (p<0.0001), female r = 0.66 (p<0.0001).

**Figure 3:**
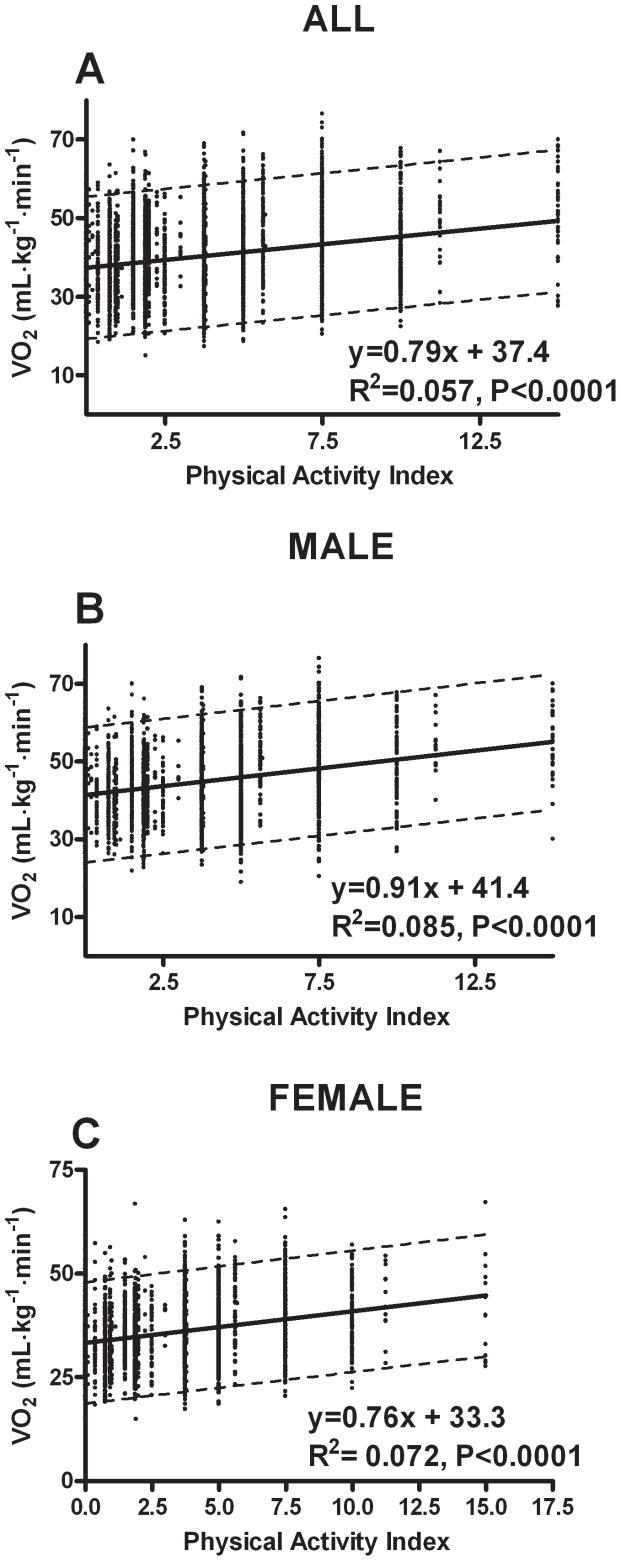
Correlations between physical activity index score and oxygen uptake (VO_2_).

Figure 3 demonstrates a poor, but statistically strong association between Physical activity index and VO_2_ (mL·kg^−1^·min^−1^); all r = 0.24 (p<0.0001), male r = 0.29 (p<0.0001) and female r = 0.27 (p<0.0001).

**Figure 4:**
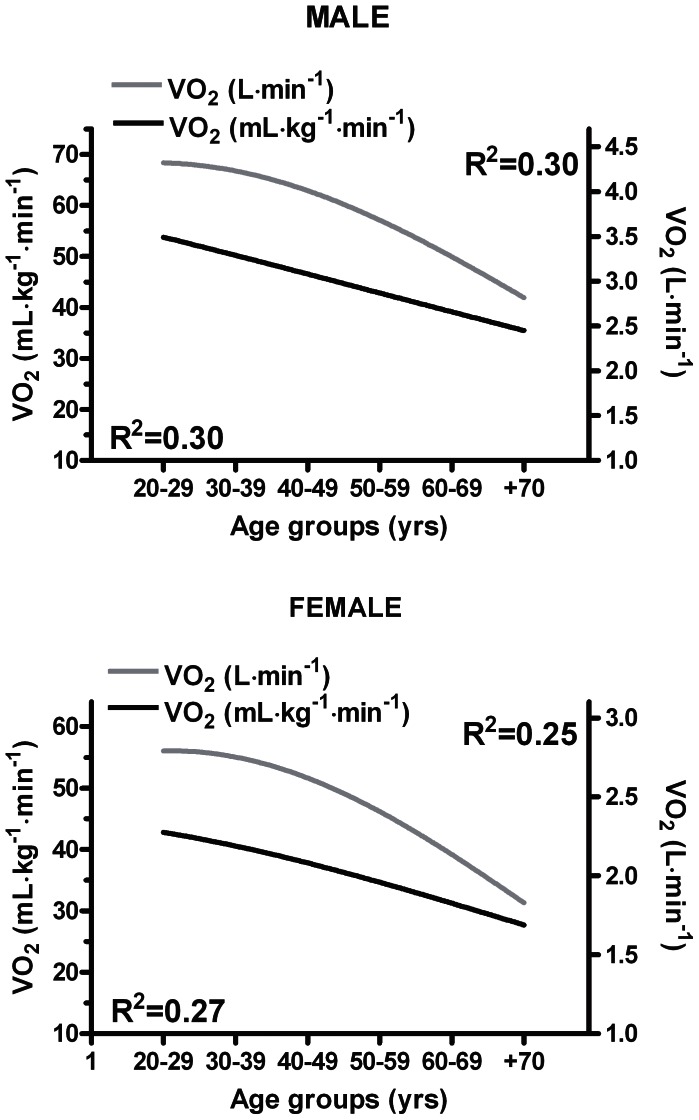
Decline of oxygen uptake (VO_2_) relative to age. Mean and SD for each age group is presented in Table 2.

Figure 4 exhibits a moderate association between VO_2_ (both mL·kg^−1^·min^−1^ and L·min^−1^) and age groups, male: r = 0.54 and r = 0.54, respectively; female: r = 0.52 and r = 0.50, respectively.

## Discussion

This is the largest European reference material of objectively measured aerobic capacity and exercise-physiology in healthy men and women aged 20–90 yrs. Our observations are an important supplement to previously published data that have mostly been either indirect or based on small, selected, or poorly-described populations [8]–[14].

### Sex Differences in VO_2max_ and Maximal Heart Frequencies

Despite being generally more physical active than men, women had a 34% and 18.5% lower absolute (L·min^−1^) and relative (mL·kg^−1^·min^−1^) VO_2max_, respectively, than men. When applying appropriate scaling procedures [27] where differences in body mass are taken into consideration for a more accurate comparison [27]–[29], women had 22.7% lower VO_2max_ (mL·kg^−0.75^·min^−1^) than men. The higher VO_2max_ in men is in accordance with former studies [5], [10]–[14], [30]. We observed higher VO_2max_ compared to that reported in American [9], [11], [13], [14], Japanese populations [8] and a large Brazilian study [31], especially in the younger age groups. Earlier Scandinavian research supports our VO_2max_ levels [25], [32], [33]. A study of Nomadic Lapps [34], who were physically active in taking care of their reindeers, observed VO_2max_ values close to our findings, and it is indicated that Roman legionnaires [35] had a VO_2max_ in the range of 50 mL·kg^−1^·min^−1^. Also contemporary hunter-gatherer societies display VO_2max_ in the range 50–65 mL·kg^−1^·min^−1^ in young male populations [36]. This supports the assumption that by living an active life, VO_2max_ in the range we display could be expected. When applying a scaled VO_2max_ [27], where differences in body weight is considered for a more precise comparison [27]–[29], the HUNT 3 fitness study still displays a considerable higher VO_2max_ (mL·kg^−0.75^·min^−1^) than North American, German and Asian studies. Scaled VO_2max_ in HUNT 3 was approximate 20% higher, considering both sexes and all age groups, than North American [9], [10], [37] and a German study [38]. However, a North American study by Jackson and colleagues [14] display only an estimated 10% lower scaled VO_2max_, compared to our findings, among both sexes in the 20–29 year age group, with diminishing differences per subsequent decade. A Japanese study [8] displays an average 14% lower scaled VO_2max_ than us, among both sexes and all age groups. The dissimilarities between our and other findings may be explained by that we measured VO_2max_ whereas most others [8], [9], [11], [14] use VO_2peak_ or estimated VO_2max_ [13], [15], and that most other populations might also lead a more sedate lifestyle than that of Scandinavians.

Despite our relatively high mean VO_2max_, 25 men and 9 women (>50 years) displayed values below 8 METs (28 mL·kg^−1^·min^−1^) and 6 METs (21 mL·kg^−1^·min^−1^), respectively. This is associated with higher all-cause mortality and cardiovascular events among healthy men and women [4]. 1% of men 50–59 years displayed METs associated with increased risk, with 4% and 11% prevalence of “increased risk MET” with each subsequent decade. Women displayed approximately half the prevalence over the same age groups. We report a somewhat higher maximal heart rate than previous studies, which could be explained by that others [10]–[12] measure peak heart rate. In line with former studies [10]–[12] there were no significant sex differences in maximal heart rate.

### Differences in VO_2max_ and Physical Activity Level Stratified by Age Group and Sex

The highest VO_2max_, in both men and women, were observed in the age groups 20–29 years. This fits with that both sexes in this age group had the highest level of physical activity compared to all other age groups. Between the two youngest age groups of both men and women (20–29 years and the 30–39 years) there was no difference in absolute VO_2max_ (L·min^−1^). However, among the 30–39 years old, body mass was 8.4% and 6.4% higher, and physical activity level 32.1% and 15.2% lower for men and women, respectively. A lower physical activity level did not influence the absolute VO_2max_ (L·min^−1^) but probably contribute to the higher body mass in those aged 30–39 years. Relative to body mass VO_2max_ (mL·kg^−1^·min^−1^) was 10% and a 7% lower among men and women aged 30–39 years compared to those in the 20–29 year age group. Thus, lower relative VO_2max_ in those aged 30–39 years old was caused by a higher body mass in our study population.

Despite similar body weights and physical activity level, a highly significant lower absolute (4.4%) and relative (3.8%) VO_2max_ was observed among men and women aged 40–49 years compared to those aged 30–39 years. In line with our findings, Sanada and colleagues [8] observed a similar drop in VO_2max_ between these age groups among healthy Japanese men. However, they observed a considerably larger “drop” in VO_2max_ with no change in body mass among women compared to our observations. Their findings are in agreement with another study of women by Jackson and colleagues [9]. A likely explanation for the lower VO_2max_ in those aged 40–49 vs. 30–39 could be a reduced level of physical activity in the oldest age group. However, our data does not support this. The Sanada [8] study shows a decrease in skeletal muscle mass with simultaneous increase in percent body fat. This would deteriorate demand properties and decrease VO_2max_. We do not know if this is the case in our study, but the drop in absolute VO_2max_ yields a reduction in supply properties, hence a reduction in VO_2max_. The reason for different “drop” in VO_2max_ among women in our study, the Sanada [8] and Jackson [9] studies is not known and warrant further studies.

Over the 3 next decades (40–69 years) the decrease in absolute and relative VO_2max_ had more than doubled (≈10% per decade), in both sexes, compared to that observed between age groups 30–39 years and 40–49 years. There were no significant differences in body mass between these age groups, with the exception of a ≈2% reduction in males between 50–59 and 60–69 years. The reduction in VO_2max_ per decade is in line with previous studies [8], [11], [14]. A significant reduction in physical activity level with increased age among women was observed in our study, with no changes in the male group. Thus, reduced physical activity level may explain reduced VO_2max_ with increased age among women but not among men. Whether this is really an age-related decline or due to that men have a tendency to over-report physical activity [39] is not known, and future investigations should aim to obtain objectively measured physical activity levels.

Between the 60–69 years and the +70 year age groups we observed the largest “drop” in absolute VO_2max_ (L·min^−1^) in both sexes (≈14.5%). Relatively VO_2max_ (mL·kg^−1^·min^−1^) were 10% and 11% lower in males and females aged +70 compared to those aged 60–69 years. The large drop in relative VO_2max_ occurred despite no significant drops in body mass and physical activity level. This suggests that the drop may be due to “age-related” adaptations in the organism. Our findings for these age groups are in line with previous studies reporting reductions in relative and absolute VO_2max_ in the range 13–25% and 12–29% [8], [10], [12], [40].

Ventilatory efficiency, EqVO_2max_, remains generally unchanged throughout the age groups; hence, it is not a factor in explaining the diminishing VO_2max_ with increasing age. However, subsequent 50 years of age O_2_-pulse displayed a steady decrease, which indirectly indicate a reducing stroke volume, hence this yield a reduction in VO_2max_.

### Differences in Maximum Heart Rate Stratified by Age Group and Sex

Maximum heart rate has regularly been estimated by an equation subtracting age from 220 beats·min^−1^, which have limited scientific merit [41]. The highest heart frequencies were found in the youngest age groups, regardless of sex. Maximal heart frequencies in men and women were 196±10 beats·min^−1^ and 194±9 beats·min^−1^, respectively, with a decline of approximately 3.5% (6 beats·min^−1^) per decade. Our maximal heart rate decline gradient is approximately half that compared to using the 220 beats·min^−1^ minus age equation, which is consistent with former research [10], [42], [43].

### Differences in Oxygen Pulse Stratified by Gender and Age Groups

The highest maximal oxygen pulse was observed in the 3 youngest age groups among men (22.3±3.6 mL·beat^−1^) and women (14.7±2.7 mL·beat^−1^), with no significant difference between these age groups. Previous reference material on oxygen pulse in healthy populations is based upon case reports [7] or small studies in athletes [44] [45] making comparison with our data complicated.

### Association between VO_2_ and Heart Rate

The benchmark studies of the relationship between submaximal VO_2_ and heart frequencies from the 50′s and 60′s have small sample size. When comparing our findings with that of Åstrand [6] there are obvious discrepancies. Compared with our observations, Åstrand reports lower heart frequencies at workloads corresponding to VO_2_ lower than 3 L·min^−1^, and higher heart frequencies at VO_2_ higher than 3 L·min^−1^. Since there are no references to sample size or gender in Åstrands data it is difficult to interpret the discrepancies between the studies. Another study by Åstrand [17] with 86 relatively well trained male and female students, agrees with our data, for the age group 20–29 years, on VO_2_ less than 3 L·min^−1^. When VO_2_ exceed 3.0 L·min^−1^ for females and 4.0 L·min^−1^ for males the present study displays lower heart frequencies than reported in the Åstrand study. A study [18] with 44 females, aged 20–65 years, displayed lower heart frequencies than we observed for VO_2_<1.5 L·min^−1^, but higher heart frequencies for VO_2_>1.5 L·min^−1^. The observed association between percent VO_2max_ and percent maximal heart rate is in agreement with previous studies [16], [18], [19] on intensities >70% of VO_2max_. At exercise intensities <70% of VO_2max_ we observed higher percent heart rate than previous studies. In our study 30% of VO_2max_ corresponded to 60% of maximal heart rate where others [16], [18], [19] have reported that this is equal to 50% of maximal heart rate. However, we use treadmill testing while the others use bicycle ergometer, which could explain the discrepancies.

### Association between Heart Rate and Watts

Comparing our results to a study by Åstrand [20] with 84 healthy males, good agreement was found for workloads >150W, whereas we observed progressively higher heart frequencies than Åstrand at lower workloads. This is also the case when comparing our results to another study of males by Åstrand [6]. Again, the inconsistency between our and Åstrand’s findings could be explain by treadmill vs. bicycle ergometer.

### Association between VO_2_ and Watts

We systematically display higher VO_2_ (L·min^−1^) at any given watt than that observed in two studies from Åstrand [17], [21]. Discrepancies seem to be caused by higher initial VO_2_ cost (L·min^−1^) in our results, while the slope gradient is in close proximity with Åstrand [17], [21]. The differences may be explained by that we applied treadmill work whereas Åstrand used a cycle ergometer.

### Association between Borg Scale Scores, % VO_2max_ and % Maximal Heart rRate

We observed a mismatch between the Borg study [22] and our findings. We observed considerably higher percent VO_2max_ and percent maximal heart rate for a given exertion interval than Borg [22], but differences vanished at the highest Borg-levels.

Relative to VO_2max_ and maximal heart rate Borg scale differed between sexes. In the range 6–15 on Borg scale males worked at a lower percent (4%) of both VO_2max_ and maximal heart rate than females, i.e. the relative rating of perceived exertion in males were higher at a given work load. There were no differences between sexes at Borg16–20. Our data clearly support the notion that Borg-scale may be used as a robust tool to guide exercise intensity in healthy men and women, but that one should be aware of sex differences at the lowest Borg levels.

### Association between Physical Activity Index Scores and VO_2max_

There was a poor overall correlation (r = 0.24) between self-reported physical activity level and VO_2_, which indicates that only 5.7% of differences in VO_2max_ can be explained by the physical activity scores. This is in agreement with prior research [11], [46], [47]. Physical activity index score (PAI) is a weighted product between duration, frequency and intensity. Intensity might be weighted to low, thus it could explain the poor correlation between PAI and VO_2_.

### Limitations

This study may be subject to bias due to self-selection caused by the low participation rate. However, almost all of those who were invited to the current Fitness study from the large HUNT study agreed to participate in the fitness test. Due to limited capacity at the test sites resulting in long waiting lines, many potential participants chose to withdraw their participation from the study. Those who finally participated in the study could thus be healthier than those who quit or declined participation. However, a comparison of the participants in the fitness study with a healthy sample of the total HUNT population (i.e. free from cardiovascular or pulmonary diseases, cancer, or sarcoidosis) confirmed that the fitness participants did not considerably differ from other healthy HUNT participants [48]. In future studies physical activity should be measured objectively rather than being self-reported, given the large inconsistencies between VO_2max_ and self-reported physical activity.

## Conclusions

The discrepancies between this and previous studies highlighted the need of a large reference material as presented in this study. The HUNT 3 Fitness study presents the largest Europen reference material of objectively measured parameters of aerobic capacity and exercise-physiology in healthy men and women aged 20–90 years. Our data establishes normal values for the key physiological factors VO_2max_ and heart rate, as well as associations between commonly used exercise parameters. The data forms the basis for a user-friendly tool for exercise intensity control in healthy men and women.

## References

[1] Erikssen G, Liestol K, Bjornholt J, Thaulow E, Sandvik L, et al (1998) Changes in physical fitness and changes in mortality. Lancet 352: 759–762. 9737279 10.1016/S0140-6736(98)02268-5

[2] Myers J, Prakash M, Froelicher V, Do D, Partington S, et al (2002) Exercise capacity and mortality among men referred for exercise testing. N Engl J Med 346: 793–801. 11893790 10.1056/NEJMoa011858

[3] Sandvik L, Erikssen J, Thaulow E, Erikssen G, Mundal R, et al (1993) Physical fitness as a predictor of mortality among healthy, middle-aged Norwegian men. N Engl J Med 328: 533–537. 8426620 10.1056/NEJM199302253280803

[4] Kodama S, Saito K, Tanaka S, Maki M, Yachi Y, et al (2009) Cardiorespiratory fitness as a quantitative predictor of all-cause mortality and cardiovascular events in healthy men and women: a meta-analysis. JAMA 301: 2024–2035. 19454641 10.1001/jama.2009.681

[5] Lee DC, Sui X, Ortega FB, Kim YS, Church TS, et al (2011) Comparisons of leisure-time physical activity and cardiorespiratory fitness as predictors of all-cause mortality in men and women. Br J Sports Med 45: 504–510. 20418526 10.1136/bjsm.2009.066209

[6] Åstrand P, Rodahl K, Dahl H, Strømme S (2003) Textbook of Work Physiology Physiological Bases of Exercise: McGraw-Hill.

[7] Wasserman K, Hansen JE, Sue DY, Stringer WS, Sietsema KE, et al.. (2012) Principles of Exercise Testing and Interpretation: Lippincott Williams &Wilkins.

[8] Sanada K, Kuchiki T, Miyachi M, McGrath K, Higuchi M, et al (2007) Effects of age on ventilatory threshold and peak oxygen uptake normalised for regional skeletal muscle mass in Japanese men and women aged 20–80 years. Eur J Appl Physiol 99: 475–483. 17186296 10.1007/s00421-006-0375-6

[9] Jackson AS, Wier LT, Ayers GW, Beard EF, Stuteville JE, et al (1996) Changes in aerobic power of women, ages 20–64 yr. Med Sci Sports Exerc 28: 884–891. 8832543 10.1097/00005768-199607000-00016

[10] Fleg JL, Morrell CH, Bos AG, Brant LJ, Talbot LA, et al (2005) Accelerated longitudinal decline of aerobic capacity in healthy older adults. Circulation 112: 674–682. 16043637 10.1161/CIRCULATIONAHA.105.545459

[11] Talbot LA, Metter EJ, Fleg JL (2000) Leisure-time physical activities and their relationship to cardiorespiratory fitness in healthy men and women 18–95 years old. Med Sci Sports Exerc 32: 417–425. 10694126 10.1097/00005768-200002000-00024

[12] Hollenberg M, Ngo LH, Turner D, Tager IB (1998) Treadmill exercise testing in an epidemiologic study of elderly subjects. J Gerontol A Biol Sci Med Sci 53: B259–267. 18314555 10.1093/gerona/53a.4.b259

[13] Wang CY, Haskell WL, Farrell SW, Lamonte MJ, Blair SN, et al (2010) Cardiorespiratory fitness levels among US adults 20–49 years of age: findings from the 1999–2004 National Health and Nutrition Examination Survey. Am J Epidemiol 171: 426–435. 20080809 10.1093/aje/kwp412

[14] Jackson AS, Sui X, Hebert JR, Church TS, Blair SN (2009) Role of lifestyle and aging on the longitudinal change in cardiorespiratory fitness. Arch Intern Med 169: 1781–1787. 19858436 10.1001/archinternmed.2009.312 PMC3379873

[15] Armstrong L, Balady GJ, Berry MJ, Davis SE, Davy BM, et al.. (2006) ACSM’s Guidelines for Exercise Testing and Prescription: Lippincott Williams and Wilkins. 79 p.

[16] McArdle W, Katch F, Katch V (2010) Exercise Physiology, Nutrition, Energy, and Human Performance. Philadelphia, PA: Lippincott Williams & Wilkins.

[17] Åstrand P (1952) Experimental studies of physical working capacity in relation to sex and age. Copenhagen: Munksgaard.

[18] Taylor H, Haskell W, Fox S, Blackburn H (1969) Measurement in Exercise Electrocardiography; Blackburn H, editor. Illinois, USA: Charles C Thomas Publisher.

[19] Pollock M, Wilmore J, Fox S (1978) Health and fitness through physical activity: John Wiley & Sons.

[20] Astrand I (1967) Degree of strain during building work as related to individual aerobic work capacity. Ergonomics 10: 293–303. 6077517 10.1080/00140136708930871

[21] Astrand PO (1976) Quantification of exercise capability and evaluation of physical capacity in man. Prog Cardiovasc Dis 19: 51–67. 785542 10.1016/0033-0620(76)90008-6

[22] Borg GA (1982) Psychophysical bases of perceived exertion. Med Sci Sports Exerc 14: 377–381. 7154893

[23] Alberton CL, Antunes AH, Pinto SS, Tartaruga MP, Silva EM, et al (2011) Correlation between rating of perceived exertion and physiological variables during the execution of stationary running in water at different cadences. J Strength Cond Res 25: 155–162. 20093964 10.1519/JSC.0b013e3181bde2b5

[24] Rognmo O, Hetland E, Helgerud J, Hoff J, Slordahl SA (2004) High intensity aerobic interval exercise is superior to moderate intensity exercise for increasing aerobic capacity in patients with coronary artery disease. Eur J Cardiovasc Prev Rehabil 11: 216–222. 15179103 10.1097/01.hjr.0000131677.96762.0c

[25] Hermansen L, Saltin B (1969) Oxygen uptake during maximal treadmill and bicycle exercise. J Appl Physiol 26: 31–37. 5762873 10.1152/jappl.1969.26.1.31

[26] Kurtze N, Rangul V, Hustvedt BE, Flanders WD (2008) Reliability and validity of self-reported physical activity in the Nord-Trondelag Health Study: HUNT 1. Scand J Public Health 36: 52–61. 18426785 10.1177/1403494807085373

[27] Batterham AM, Birch KM (1996) Allometry of anaerobic performance: a gender comparison. Can J Appl Physiol 21: 48–62. 8664846 10.1139/h96-005

[28] Chamari K, Moussa-Chamari I, Boussaidi L, Hachana Y, Kaouech F, et al (2005) Appropriate interpretation of aerobic capacity: allometric scaling in adult and young soccer players. Br J Sports Med 39: 97–101. 15665205 10.1136/bjsm.2003.010215 PMC1725118

[29] Helgerud J (1994) Maximal oxygen uptake, anaerobic threshold and running economy in women and men with similar performances level in marathons. Eur J Appl Physiol Occup Physiol 68: 155–161. 8194545 10.1007/BF00244029

[30] Macek M, Seliger V, Vavra J, Skranc O, Horak J, et al (1979) Physical fitness of the Czechoslovak population between the ages of 12 and 55 years. Oxygen consumption and pulse oxygen. Physiol Bohemoslov 28: 75–82. 155833

[31] Herdy AH, Uhlendorf D (2011) Reference values for cardiopulmonary exercise testing for sedentary and active men and women. Arq Bras Cardiol 96: 54–59. 21109909 10.1590/s0066-782x2010005000155

[32] Hermansen L, Andersen KL (1965) Aerobic work capacity in young Norwegian men and women. J Appl Physiol 20: 425–431. 5319990 10.1152/jappl.1965.20.3.425

[33] Andersen LB, Henckel P, Saltin B (1987) Maximal oxygen uptake in Danish adolescents 16–19 years of age. Eur J Appl Physiol Occup Physiol 56: 74–82. 3104033 10.1007/BF00696380

[34] Lange-Andersen L, Elsner R, Saltin B, Hermansen L (1961–1962) Physical fitness in terms of maximal oxygen uptake of nomadic lapps. Arctic Aeromedical Laboratory, Alaska.

[35] Mitchell JH, Saltin B (2003) The oxygen transport system and maximal oxygen uptake. New York: Oxford University press.

[36] Cordain L, Gotshall RW, Eaton SB, Eaton SB 3rd (1998) Physical activity, energy expenditure and fitness: an evolutionary perspective. Int J Sports Med 19: 328–335. 9721056 10.1055/s-2007-971926

[37] Nelson MD, Petersen SR, Dlin RA (2010) Effects of age and counseling on the cardiorespiratory response to graded exercise. Med Sci Sports Exerc 42: 255–264. 19927033 10.1249/MSS.0b013e3181b0e534

[38] Meyer K, Hajric R, Samek L, Baier M, Lauber P, et al (1994) Cardiopulmonary exercise capacity in healthy normals of different age. Cardiology 85: 341–351. 7850824 10.1159/000176733

[39] Klesges RC, Eck LH, Mellon MW, Fulliton W, Somes GW, et al (1990) The accuracy of self-reports of physical activity. Med Sci Sports Exerc 22: 690–697. 2233209 10.1249/00005768-199010000-00022

[40] Weiss EP, Spina RJ, Holloszy JO, Ehsani AA (2006) Gender differences in the decline in aerobic capacity and its physiological determinants during the later decades of life. J Appl Physiol 101: 938–944. 16497840 10.1152/japplphysiol.01398.2005

[41] Robergs R, Landwehr R (2002) The surprising history of the “HRmax = 220-age” Equation. Journal of Exercise Physiology online 5.

[42] Tanaka H, Monahan KD, Seals DR (2001) Age-predicted maximal heart rate revisited. J Am Coll Cardiol 37: 153–156. 11153730 10.1016/s0735-1097(00)01054-8

[43] Gellish RL, Goslin BR, Olson RE, McDonald A, Russi GD, et al (2007) Longitudinal modeling of the relationship between age and maximal heart rate. Med Sci Sports Exerc 39: 822–829. 17468581 10.1097/mss.0b013e31803349c6

[44] Sharma S, Elliott PM, Whyte G, Mahon N, Virdee MS, et al (2000) Utility of metabolic exercise testing in distinguishing hypertrophic cardiomyopathy from physiologic left ventricular hypertrophy in athletes. J Am Coll Cardiol 36: 864–870. 10987612 10.1016/s0735-1097(00)00816-0

[45] Padilla P, Ojeda C, Fernandez C, Licea M (2000) Maximum oxygen pulse in high performance mexican athletes. Rev Inst Nal Enf Resp Mex 13: 73–84.

[46] van Poppel MN, Chinapaw MJ, Mokkink LB, van Mechelen W, Terwee CB (2010) Physical activity questionnaires for adults: a systematic review of measurement properties. Sports Med 40: 565–600. 20545381 10.2165/11531930-000000000-00000

[47] Tager IB, Hollenberg M, Satariano WA (1998) Association between self-reported leisure-time physical activity and measures of cardiorespiratory fitness in an elderly population. Am J Epidemiol 147: 921–931. 9596470 10.1093/oxfordjournals.aje.a009382

[48] Aspenes ST, Nilsen TI, Skaug EA, Bertheussen GF, Ellingsen O, et al (2011) Peak oxygen uptake and cardiovascular risk factors in 4631 healthy women and men. Med Sci Sports Exerc 43: 1465–1473. 21228724 10.1249/MSS.0b013e31820ca81c

