# Correction: Aerobic Capacity Reference Data in 3816 Healthy Men and Women 20–90 Years

**DOI:** 10.1371/annotation/e3115a8e-ca9d-4d33-87ef-f355f07db28e

**Published:** 2013-11-07

**Authors:** Henrik Loe, Øivind Rognmo, Bengt Saltin, Ulrik Wisløff

In Table 2 of the PDF, variables under the table heading 50-59 years were erroneously listed under 20-29 years. These variables should remain under 50-59 years. Please see the corrected Table 2 here: http://www.plosone.org/corrections/pone.0064319.t002.tif

